# “Excess” electrons in LuGe

**DOI:** 10.1002/anie.202014284

**Published:** 2021-01-26

**Authors:** Riccardo Freccero, Julia‐Maria Hübner, Yurii Prots, Walter Schnelle, Markus Schmidt, Frank R. Wagner, Ulrich Schwarz, Yuri Grin

**Affiliations:** ^1^ Abteilung Chemische Metallkunde Max-Planck-Institut für Chemische Physik fester Stoffe Nöthnitzer Str. 40 01187 Dresden Germany

**Keywords:** chemical bonding, germanium, high-pressure synthesis, intermetallic compound, lutetium

## Abstract

The monogermanide LuGe is obtained via high‐pressure high‐temperature synthesis (5–15 GPa, 1023–1423 K). The crystal structure is solved from single‐crystal X‐ray diffraction data (structure type FeB, space group *Pnma*, *a*=7.660(2) Å, *b*=3.875(1) Å, and *c*=5.715(2) Å, R_F_=0.036 for 206 symmetry independent reflections). The analysis of chemical bonding applying quantum‐chemical techniques in position space was performed. It revealed—beside the expected 2c‐Ge‐Ge bonds in the germanium polyanion—rather unexpected four‐atomic bonds between lutetium atoms indicating the formation of a polycation by the excess electrons in the system Lu^3+^(2b)Ge^2−^×1 e^−^. Despite the reduced VEC of 3.5, lutetium monogermanide is following the extended 8‐N rule with the trend to form lutetium‐lutetium bonds utilizing the electrons left after satisfying the bonding needs in the anionic Ge‐Ge zigzag chain.

## Graphical abstract

The germanium monogermanide is prepared by high‐pressure, high‐temperature synthesis. Chemical bonding in LuGe is described as covalently bonded zigzag chains of Ge atoms with the counterpart Lu_4_ polycations formed using excess electrons in sense of the Zintl count Lu^3+^(2b)Ge^2−^×1 e^−^. The homonuclear bonding in polycation in Lu_4_Ge_4_, which makes use of the electrons not required in the polyanion, resembles the situation in Ga_2_Se_2_.

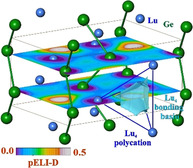

The structures of FeB^[1]^ and α‐TlI,^[2]^ first investigated in the 30ies, were considered as characteristic examples of “intermetallic” (FeB with trigonal prismatic coordination of the smaller boron atoms, CN=9) or ionic (α‐TlI as a distorted variant of the NaCl type) compounds. The later characterisation of the boride CrB^[3]^ revealed that the structural pattern of α‐TlI is also well suitable for typical intermetallic compounds of elements with much lower difference in electronegativity. The discovery of the first monotetrelide—CaSi^[4]^—revealed, that the same structural pattern is compatible with covalent bonding in the chain anion of two‐bonded silicon atoms plus its ionic interaction with the cations in accordance with the Zintl‐Klemm concept: Ca^2+^(2b)Si^2−^.[^5^, ^6^, ^7^] The subsequent finding of the monotetrelides YSi^[8]^ or LaSi^[9]^ evidenced that chains of two‐bonded tetrel atoms can also form in presence of “excess” electrons, for example, Y^3+^(2b)Si^2−^×1 e^−^. Recent quantum chemical studies on La_2_ *M*Ge_6_ (*M*=Li, Mg, Al, Zn, Cu, Ag, Pd) and Y_2_PdGe_6_ comprising similar zig‐zag chains revealed further significant deviations from the formal 8‐*N* picture due to polar‐covalent interactions.^[10]^

Systematic investigations going back to the 70ies revealed the new chain anion (2b)Si^2−^ in a second modification of LaSi.^[11]^ Strontium monosilicide with a new band‐like anion of (2b)Si^2−^ and (3b)Si^1−^ ^[12]^ completed the series of monotetrelides with rare‐earth and alkaline metals compounds.^[13]^ Thereby, high pressure‐high temperature (HP‐HT) preparation was found to be a very efficient tool for the preparation of new modifications of the members of this family and new intermetallic phases in general.[^14^, ^15^] Among the monogermanides, the representatives of the heavy rare‐earth metals are less studied. This was the reason to apply the HP‐HT technique for the preparation of LuGe, which is not known in the published phase diagram.^[16]^

The product of the HT‐HP preparation is brittle bulk with dark metallic luster. The compound is a high‐pressure phase. By heating under ambient pressure up to 873 K, the structure keeps intact, but the lattice starts to lose one of the components, which leads to the marked reduction of the lattice parameters *a*=7.735(1) Å, *b*=3.8579(5) Å, *c*=5.630(1) Å (cf. below). After further heating in a DSC apparatus up to 1023 or 1323 K, only the thermodynamically stable phases Lu_3_Ge_4_ and Lu_11_Ge_10_ are present in the sample, but no clear decomposition effect was detected. Further DSC experiment up to 1673 K reveals an endothermal effect at 1595(10) K corresponding to the peritectoid decomposition of Lu_3_Ge_4_ into *ht*‐Lu_2_Ge_3_ and Lu_11_Ge_10_, reported in the phase diagram.^[16]^

The powder X‐ray diffraction pattern of LuGe is indexed in the orthorhombic system with *a*=7.660(2) Å, *b*=3.875(1) Å, and *c*=5.715(2) Å. Further characterization of the crystal structure was performed on basis of single crystal diffraction data (crystallographic information is presented in Table S1 in the Supporting Information). Systematic extinctions yielded possible space groups *Pnma* and *Pn*2_1_ *a*, agreeing with the supposed structure of FeB type. The refinement in the centrosymmetric space group resulted in residuals *R*=0.0360 and *wR*=0.0455 denoting a sound agreement of structure model and data. The final atomic coordinates and displacement parameters are presented in Table S1, the anisotropic displacement parameters and interatomic distances can be found in Tables S2 and S3. The occupation factors of lutetium and germanium were refined to unity within the error margin, yielding the equiatomic composition LuGe. The composition Lu_51.5(5)_Ge_48.5(5)_ found by EDXS measurements is in fair agreement (within 3 e.s.d.) with the results of the structure refinement.

The new phase is isotypic to FeB.^[1]^ In the crystal structure of LuGe, each lutetium atom possesses seven germanium and ten lutetium neighbors. Germanium is coordinated by two germanium and seven lutetium atoms (Table S3). The crystal structure is constituted by columns of triangular prisms [GeLu_9_] condensed along [010] by rectangular faces (Figure 1, top).^[17]^ Within these columns, zigzag chains of interconnected germanium atom run along the [010] direction. The distances *d*(Ge‐Ge) amount to 2.594(2) Å, the distances *d*(Lu‐Ge) range between 2.897(1) and 3.168(3) Å, the shortest *d*(Lu‐Lu) amounts to 3.609(1) Å. The neighboring columns share edges along [010]. The space between the columns is filled by empty distorted tetrahedra [Lu_4_] and half‐octahedra [Lu_5_] (Figure 1, bottom), which is also observed in the α‐ITl‐ and LaSi‐type monotetrelides. This is a more general feature of intermetallic compounds with trigonal‐prismatic coordination.^[18]^

**Figure 1:**
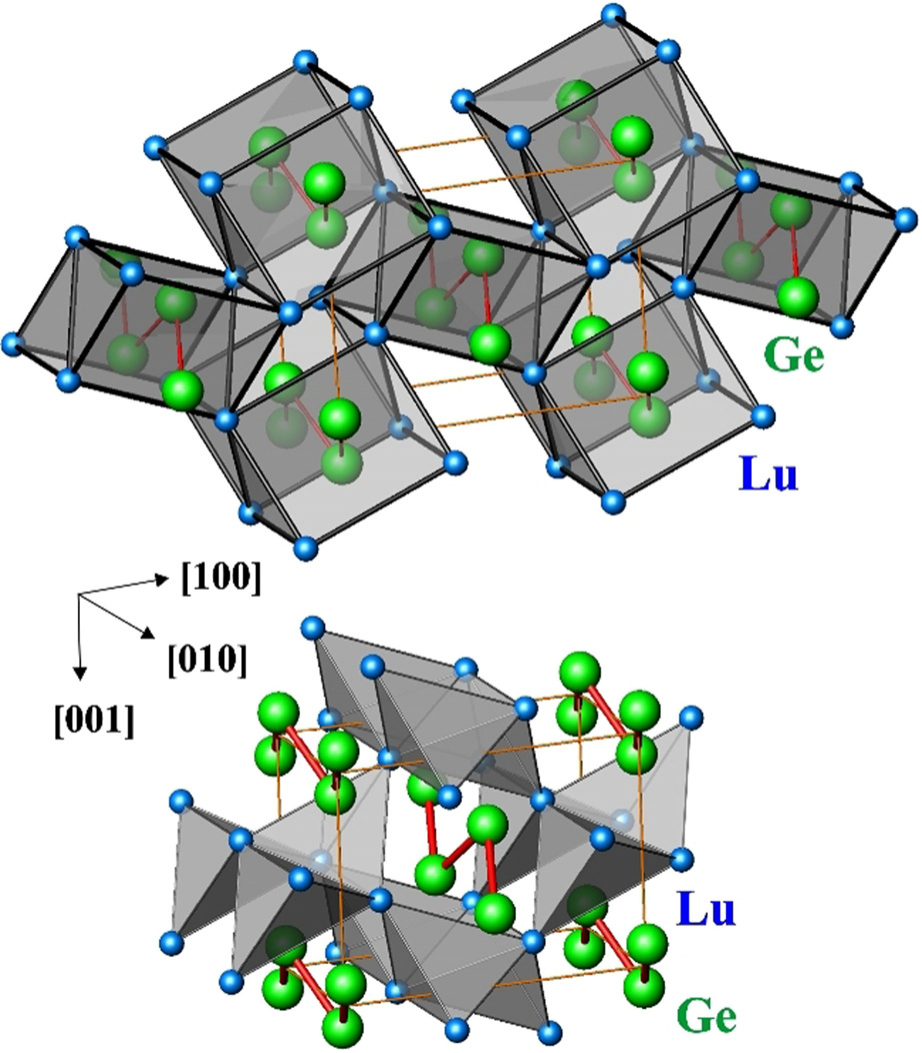
Crystal structure of LuGe: (top) zig‐zag Ge chains inside the columns of face‐condensed trigonal prisms [Lu_6_]; (bottom) empty tetrahedra [Lu_4_] between the trigonal prisms. Red lines denote the shortest Ge‐Ge distances, orange lines show the unit cell.

Other alkaline‐earth and rare‐earth metal monogermanides crystallize in the structure types α‐TlI, FeB, SrSi or LaSi, respectively. With the exception of the germanium‐deficient compound SrGe_0.76_ adopting the SrSi‐type structure,^[12]^ these structure types comprise Ge‐Ge chains. The adaption of the structure and the chains itself upon implementation of different alkaline‐earth and rare‐earth metal atoms is illustrated by the examination of the average atomic volume *V̄*_A_ (unit cell volume divided by number of atoms per unit cell) and the distances *d*(Ge‐Ge). While the atomic volume increases with the size of the cation (Figure 2), the chain distances *d*(Ge‐Ge) in the monogermanides scatter strongly and are significantly longer than the contacts in elemental Ge (2.449 Å). The elongation of homoatomic contacts in monotetrelides in comparison with the α‐modifications of the according elements was discussed in different ways. From the reconstructed electron density experiments on CaSi (structure type α‐TlI), the elongated distances Si‐Si (and the metallic behavior) are suggested to originate from the delocalized distribution of the valence electron density over the germanium chain, caused by partially covalent interactions of calcium with silicon.^[19]^ Later quantum chemical calculation on this compound revealed, that the partial occupation of π* orbitals (Si−Si bonds) reduces the charge transfer from Ca to Si and stabilizes the planarity of the Si chain,^[20]^ cf. also Supporting Information S4. A similar marked reduction of the charge transfer was observed for LaGe (structure type FeB), caused by the participation of *d* electrons in the bonding according to La^2+*d*^(2b)Ge^2−^×*d* e^−^.^[21]^

**Figure 2:**
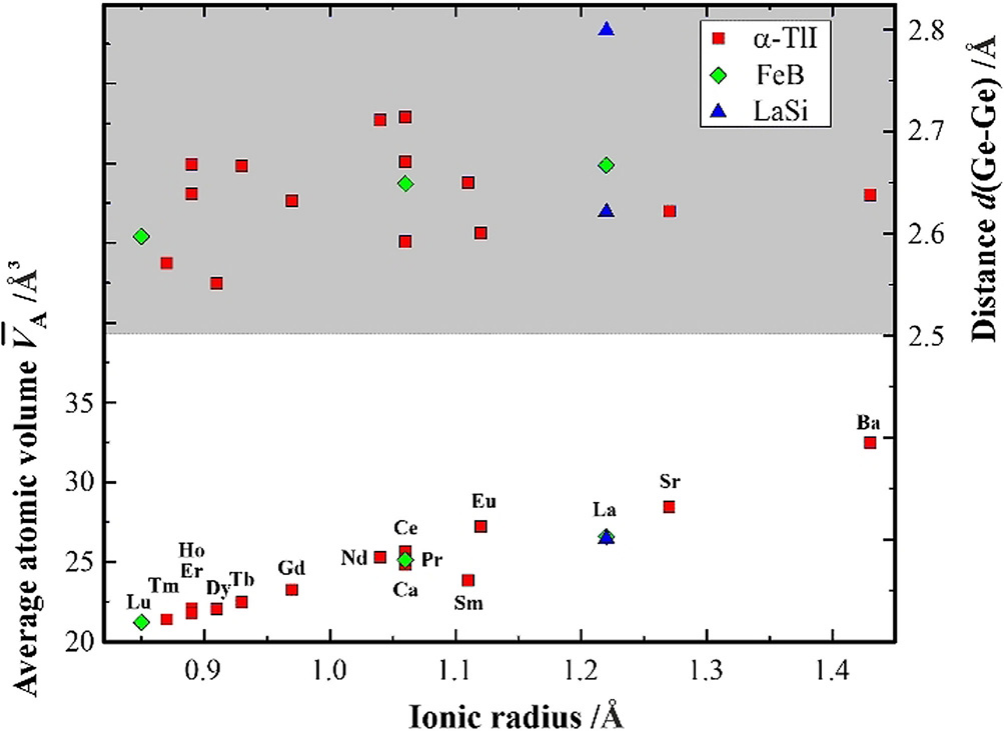
Average atomic volumes (bottom panel) and interatomic intra‐chain distances *d*(Ge‐Ge) (top panel) vs. ionic radius of the *R* ^3+^ or *M* ^2+^ cations in alkaline‐earth and rare‐earth metal monogermanides with structure types α‐TlI, FeB and LaSi.

The chains of two‐bonded tetrel atoms (2b)*Tt* are traditionally well understood within the Zintl‐Klemm concept in terms of the formal species (2b)*Tt* ^−2−^. Being two‐bonded, they need two additional electrons to fulfill the octet rule. This can be easily done in the binary monotetrelides *MTt* with *M* being an alkaline‐earth metal.[^5^, ^6^, ^7^] In these compounds, the 1:1 composition is electronically balanced, e.g., Ca^2+^(2b)Ge^2−^. Recently, this approach was found to be basically in agreement with the results of the reconstruction of electron density for CaSi from X‐ray diffraction data.^[19]^ However, the authors pointed out that the Ca‐Si interaction cannot be considered as fully ionic. The situation becomes even more intriguing for the monogermanide LuGe, in which the cation can formally offer more than two valence electrons for bonding. The electronic balance Lu^3+^(2b)Ge^2−^×1 e^−^ may be interpreted as an example of metallic behavior, where the excess electrons are filling Ge‐Ge π‐antibonding states above the pseudo gap. Another situation may appear, when the electronegativity of the cationic component allows to form homoatomic bonds employing excess electrons. Such a bonding situation may be adopted by gallium monoselenide (in a fully ionic representation) with a gallium di‐cation: [(1b)Ga^2+^]_2_[(0b)Se^2−^]_2_. In reality, the cation is not completely isolated, because of the polar covalent interactions of gallium and selenium (insufficient electronegativity difference for ionic bonding). A further possibility for the bonding use of excess electrons is the formation of a lone pair at the cation, like in indium monobromide: [(lp)In^1+^] [(0b)Br^1−^] (structure type α‐TlI). In case of full transfer of three electrons from Lu to germanium one may expect also the formation of Ge dumbbells according to [La^3+^]_2_[(1b)Ge^3−^]_2_. Such considerations were the starting point for the analysis of chemical bonding in LuGe applying quantum chemical techniques in position space.

The effective charges of the atomic species in LuGe were evaluated from the calculated electron density. First, the zero‐flux surfaces in the gradient vector field of the electron density were determined. They form the boundaries of electron density basins which represent atomic regions within the framework of the Quantum Theory of Atoms in Molecules (QTAIM^[22]^). Already the shapes of the QTAIM Lu atoms in LuGe reveal some characteristic features (Figure 3, top). Usually, the rare‐earth metals atoms in the QTAIM representation in intermetallic compounds have close to spherical shapes including mostly the inner electronic shells. The spherical shape of the yttrium cation in the recently described crystal structure of Y_4_Be_33_Pt_16_ ^[23]^ may serve as a characteristic example. In contrast, the shape of the Lu atoms in LuGe is far from spherical, indicating an appearance of rather unusual atomic interactions. The shape of the QTAIM Ge atoms is rather characteristic for a covalently bonded p‐block atom: it has plane faces toward the neighboring germanium and slightly convex surfaces toward lutetium neighbours. Then the electron density was integrated in spatial regions, defined in QTAIM, and their electronic populations yield the QTAIM effective atomic charges. The obtained charge transfer of 1.34 electron per atom (Figure 3, top panel and Table S5) is rather low in comparison with the formal charges of +3 and −3 assumed for Lu and (1b)Ge and suggests a smaller ionic contribution to the bonding in this compound in comparison with the expected one from the formal charges.

**Figure 3:**
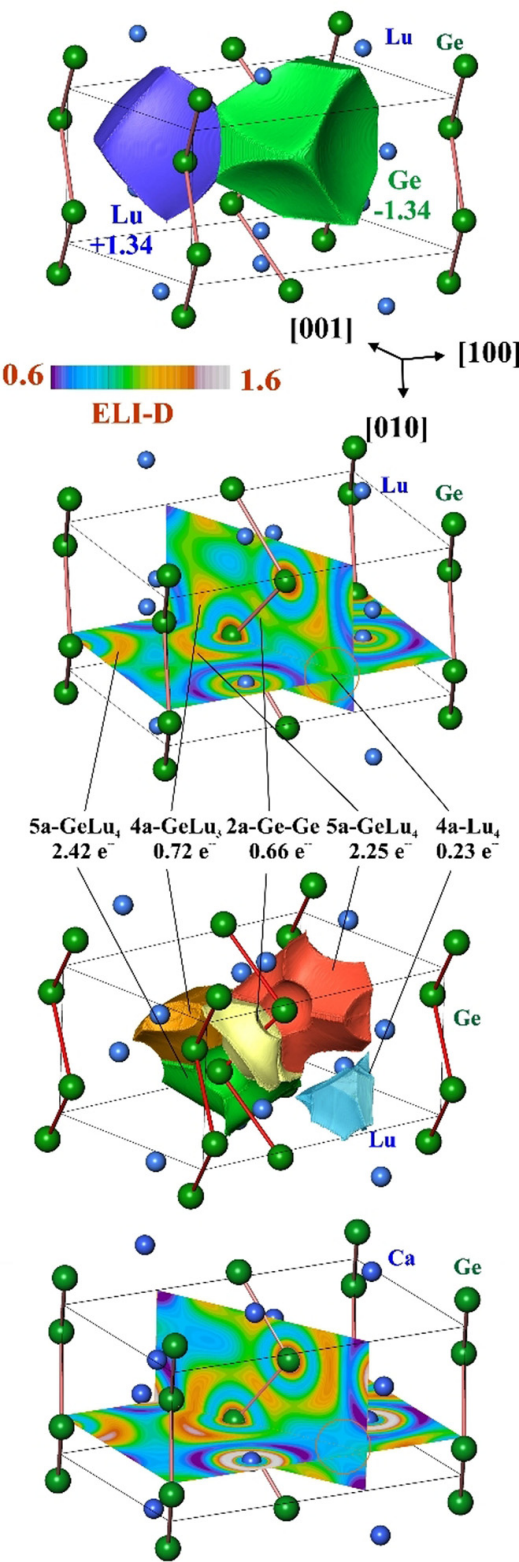
Atomic interactions in LuGe from bonding analysis in position space: (top panel) shapes and effective charges of the QTAIM atoms (atomic basins); (middle upper panel) distribution of the electron localizability indicator in the plane of the Ge chain and the (040) plane; (middle lower panel) shapes and populations of the ELI‐D bonding basins; (bottom panel) distribution of the electron localizability indicator in the hypothetic CaGe (structure type FeB) in the plane of the Ge chain and the (040) plane. The orange circle emphasizes the region of the metal‐metal interaction in LuGe and its absence in CaGe.

Further information about the atomic interactions was obtained by applying the electron localizability approach. The distribution of the electron localizability indicator (ELID) in the vicinity of the Lu nuclei (Figure 3, upper middle panel) deviates only slightly from a spherical one in the regions of the inner shells (structuring index *ϵ*=0.01^[24]^), indicating a rather small participation of the inner electrons in the bonding events. The last shell is not visible, agreeing with the charge transfer found within the QTAIM considerations. The four types of ELI‐D attractors observed in the valence region visualize different components of bonding in LuGe. The first one (the basin is shown in yellow in Figure 3, bottom) represents homoatomic Ge‐Ge bonding within the chain. The population of the bonding basin of 0.66 electrons is rather small (“1/3‐bond”, cf. interatomic distances above). The large bonding basins above and below the Ge‐chain plane (red and green in Figure 3) may be understood in first approximation as lone‐pair‐like for an isolated Ge chain. In LuGe, they are formed by contributions of one Ge and four Lu, that is, they illustrate five‐atomic polar interactions (populations of 2.25 and 2.42 electrons, respectively). The next basin (orange in Figure 3, bottom; population 0.72 electrons) can be seen as a split part of a former lone‐pair basin in the isolated chain and visualize the four atomic interaction GeLu_3_. Quantitative evaluation of the basins above (using the criteria for the position‐space characterization of the polar bonding[^25^, ^26^]) evidences that they have roughly equivalent lone‐pair‐on‐germanium and Ge‐Lu‐bonding character (Table S6). Most interesting is the presence of the last type of bonding attractors (blue basin in Figure 3, bottom; population of 0.23 electrons). They are located within the distorted tetrahedra formed by four Lu atoms (cf. Figure 1), and have common surfaces with their core basins, i.e., they are four‐synaptic. More detailed analysis by the basin‐intersection technique reveals that the majority −0.16 of the total 0.23 electrons—is contributed by the lutetium atoms, and the remaining 0.07—by four germanium atoms. Neglecting this rather small contributions of ca. 0.02 electron per one Ge would lead to the conclusion that the bonding basin represents a four‐atomic homonuclear lutetium interaction. The four‐ and six‐atomic metal‐metal interactions located at the tetrahedral and octahedral voids of the atomic pattern was already described for elementaL s‐, p‐ and d‐metals,[^27^, ^28^] and confirmed by calculations for elemental lutetium and lanthanum on the same DFT level as LuGe (Figures S7 and S8). This multi‐atomic bonding interpretation in positions space is in its main idea similar to the model of cage orbitals.[^29^, ^30^, ^31^]

The bonding picture in LuGe fits well into the series of monogermanides of alkaline‐earth and rare‐earth metals. On the other hand, it differs clearly from the bonding mechanism in more ionic representatives of these structure type, that is, the electron‐balanced α‐TlI (VEC=5) or the recently investigated electron‐deficient CaAg (VEC=1.5)^[32]^ (cf. Supporting Information S9).

From the conceptual point of view, the bonding situation in LuGe is related to the one in GaSe: the excess electrons are used for the formation of cation‐cation bonds. There is yet an important difference. While, with VEC=4.5, gallium selenide follows the Pearson’ extended 8‐*N* rule, LuGe with VEC=3.5 is following an extension of the 8‐*N* rule under “electron‐deficiency” conditions (VEC<4),^[33]^ where for each anion less than 8 electrons are accessible (available). Nevertheless, the tendency is the same: not all available electrons are required for the bonding in the germanium polyanion and—consequently—they are utilized for lutetium‐lutetium bonding.

The bonding interpretation above goes well along with the calculated electronic density of states of LuGe (Figure 4, top). It contains three well separated regions. The first one (*E* < −6 eV) contains mainly of the Ge‐s and Lu‐s states with small contributions of Ge‐p. The second one (−6 eV < *E* < −1.3 eV) has the localized Lu‐f states at its bottom and is mainly formed by the Ge‐p with admixture of Lu‐d states. A pseudo‐gap separates this region from the third one (−1.3 eV < *E* < *E*_F_). The latter is formed mainly by Lu‐d with admixture of Ge‐p states and contains ca. 4 electrons per unit cell. The first two regions represent the bonding within the Ge‐Ge zigzag chains (Ge‐Ge bonds and “lone pairs” at Ge). The calculation of partial ELI‐D^[24]^ using the states from the third region reveals, that they contribute to the ELI‐D distribution in the region of the 4a‐Lu_4_ basin representing the lutetium‐lutetium bonding. On the other hand, the same region is obviously defining the metallic behavior of LuGe, as this is confirmed by its diamagnetic behavior (Figure S10).

**Figure 4:**
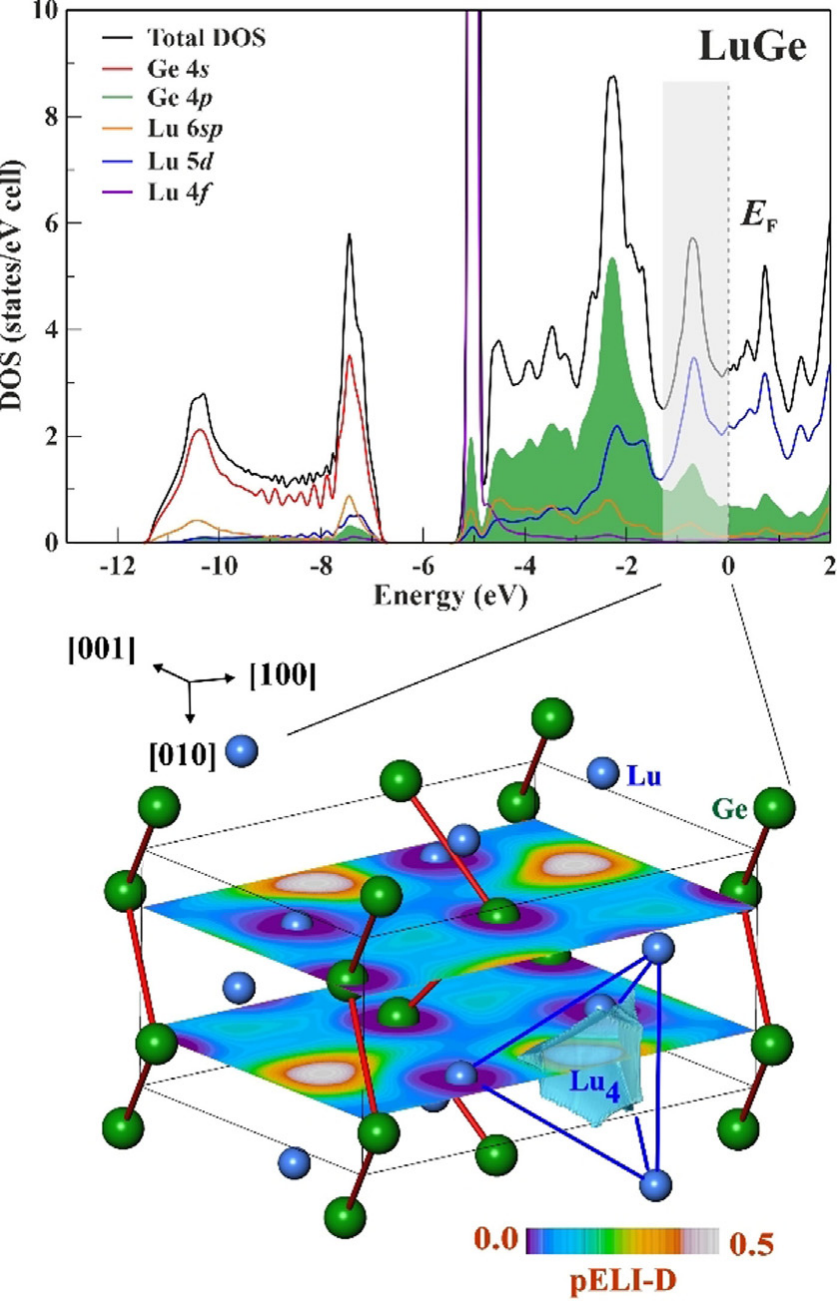
Multi‐atomic Lu interaction in LuGe: (top) total electronic density of state together with atomic contributions; (bottom) distribution of the partial ELI‐D calculated for the energy range −1.3 eV < *E* < *E*_F_ in the (*x* 1/4 *z*) and (*x* 3/4 *z*) planes revealing the lutetium contributions to the multiatomic 4*a*‐Lu_4_ interaction.

The germanium monogermanide LuGe is prepared by high‐pressure, high‐temperature synthesis. In total, the bonding in LuGe can be summarized as covalently bonded zigzag chains of Ge atoms separated from the Lu environment by multi‐atomic (four‐ and five‐atomic) bonds (originating from the lone‐pairs in an isolated chain). The lutetium atoms form four‐atomic polycations by homoatomic interactions. In such way the bonding situation in LuGe is similar to that in GaSe: the interaction in the polycation is a four‐atomic one in LuGe and two‐atomic in the selenide; the germanium atoms form a chain polyanion, while isolated Se anion can be found in GaSe.

## Conflict of interest

The authors declare no conflict of interest.

## Supporting information

As a service to our authors and readers, this journal provides supporting information supplied by the authors. Such materials are peer reviewed and may be re‐organized for online delivery, but are not copy‐edited or typeset. Technical support issues arising from supporting information (other than missing files) should be addressed to the authors.

Supplementary
