# Supplementary material for: “Excess” electrons in LuGe
Source: Angew Chem Int Ed Engl. 2021 Jan 26;60(12):6457–61. doi: 10.1002/anie.202014284 (PMC7986909; doi:10.1002/anie.202014284)
Supplement: Supplementary file 1 — Supplementary [file ANIE-60-6457-s001.pdf]

Angewandte Chemie
Eine Zeitschrift der Gesellschaft Deutscher Chemiker

Supporting Information

“Excess” electrons in LuGe

Riccardo Freccero, Julia-Maria Hübner, Yurii Prots, Walter Schnelle, Markus Schmidt, Frank R. Wagner, Ulrich Schwarz,* and Yuri Grin**

anie_202014284_sm_miscellaneous_information.pdf

Experimental Section

LuGe is obtained by high-pressure, high-temperature synthesis between 5(1) GPa and 15(2) GPa and temperatures from 1023(102) to 1423(142) K before quenching under load. Phase designation and least-squares refinement of lattice parameters were performed on basis of powder X-ray Guinier diffraction data (Cu$K_{\alpha 1}$ radiation, λ = 1.54056 Å, graphite monochromator, Huber 670 camera, $5° \leq 2\theta \leq 100°$, $\Delta\ 2\theta$ = 0.005°) at room temperature with LaB_6 (NIST) as an internal standard. Single crystal X-ray diffraction was conducted with a Rigaku Saturn724+ diffractometer (CCD detector, MoK_{α} radiation, λ = 0.71073 Å). Absorption correction was done by a multi-scan procedure. All crystallographic calculations were performed with the WinCSD program package [34].

Differential scanning calorimetry (DSC) experiments were conducted in a Netzsch DSC 404C device (Netzsch-Gerätebau GmbH, Selb, Germany) by using corundum crucibles and heating and cooling rates of 10 K min^{-1} under argon atmosphere. Energy-dispersive X-ray spectroscopy (EDXS) was realized with a Philips XL 30 scanning electron microscope (LaB_6 cathode) and an attached EDAX Si(Li) detector. Magnetic susceptibility measurements were performed on polycrystalline samples of cylindrical shape (3 × 3 × 2 mm) with a SQUID magnetometer (MPMS XL-7, Quantum Design) between 1.8 and 300 K in external fields of 0.01 to 3.5 T.

The experimentally determined structure of LuGe was optimized employing the all-electron FHI-aims [35] program at the DFT/PBE [36] level of theory and using for both atomic species the predefined default “tight” basis sets. The Brillouin zone was sampled with a 4×8×4 *k*-point mesh. Scalar relativistic effects for all electrons were considered within the ZORA approximation. The position-space chemical bonding analysis was performed using the obtained wave function. The calculation of the electron density and the electron localizability indicator (ELI-D [24,37]) effectuated on an equidistant grid of about 0.05 bohr mesh size. Their topological analysis (based on the QTAIM theory [22]) was performed with the program DGrid [38]. A PSC0 (penultimate shell correction of lowest order [10]) correction of the penultimate shell’s average electronic populations was applied. The Ge^{4+} and Lu^{3+} atomic core charges were chosen. The same technique was applied for the calculations on the elemental Lu and La, the hypothetic CaGe (FeB type), LaGe and LuGe (α-TlI type), as well as the existing LaGe (FeB type) and CaGe (α-TlI type).

Table S1. Crystallographic information for LuGe.

Composition	LuGe
Space group, Pearson symbol	*Pnma* (No. 62), *oP*8
Structure type	FeB
Unit cell parameters (PXRD)	
a, Å	7.660(2)
b, Å	3.875(1)
c, Å	5.715(2)
V, Å^3	169.6(2)
Formula units per unit cell	4
Diffraction experiment	Rigaku AFC7 diffractometer, Saturn724+ CCD detector, graphite monochromator, Mo$K\alpha$ radiation, λ = 0.71073 Å
Reflections collected, symmetry independent, used for refinement with $F > 4\sigma(F)$	1297, 311, 254
Index range measured	$-11 \le h \le 10$, $-5 \le k \le 5$, $-3 \le l \le 8$
Residuals	R = 0.037, wR = 0.039, GOF = 1.04
Atoms	
Lu in 4*c* (x¼z): x, z, B_{eq} (Å^2)	0.1784(1); 0.6174(2); 0.97(2)
Ge in 4*c* (x¼z): x, z, B_{eq} (Å^2)	0.0411(3); 0.1405(4); 1.18(5)

Table S2. Anisotropic displacement parameters in LuGe.

Atom	B_{11}	B_{22}	B_{33}	B_{12}	B_{13}	B_{23}
Lu	1.08(4)	1.09(4)	0.73(4)	0	0.09(3)	0
Ge	0.78(8)	1.34(8)	1.41(8)	0	-0.03(7)	0

Table S3. Interatomic distances in LuGe.

Atoms	Distance / Å	Atoms	Distance / Å
Lu – 2 Ge	2.897(2)	Ge – 2 Ge	2.594(2)
– 2 Ge	2.914(2)	– 2 Lu	2.897(1)
– 1 Ge	2.922(3)	– 2 Lu	2.914(2)
– 1 Ge	3.145(3)	– 1 Lu	2.922(3)
– 1 Ge	3.169(3)	– 1 Lu	3.145(3)
– 2 Lu	3.609(1)	– 1 Lu	3.168(3)
– 4 Lu	3.623(1)		
– 2 Lu	3.8750(1)		
– 2 Lu	4.119(2)		

The idea, that true (2b)E species display a spiral partial structure and not a plane one, has been discussed in literature. However, such a claim goes clearly beyond the 8-N rule, since it concerns the secondary structure of a polymer. If we consider not only spiral Se chains, but also cyclic S_8 molecules as a realization of the classical covalent bonding rules we approach the "problem" of distinguishing between the chair and the boat form of cyclohexane, one being related to classical bonding concepts, and the other one not. So, we consider the 1D-zig-zag chain as a conceptually included case for (2b)*E* species.

Also, in α-ITl(CrB)-type CaGe, LaGe, and LuGe, there may be a conceptual problem with the incompletely filled p_z states of Ge leading to a certain pi-bonding character within the chain. But this overlaps with another effect that has been often underestimated - the formation of polar covalent M–Ge or R–Ge bonds due to incomplete charge transfer. A recent detailed investigation of this kind of effect in La_2MGe_6 compounds [10] with (2b)Ge and (3b)Ge chains showed, the so-called pi states, which are displayed in the ELI-D representation as two lone-pair-type features in pseudo-tetrahedral directions clearly disfavor the comparison with the trans-polyacetylene chain, as interesting as this idea may be. Instead, significant polar-covalent interactions M–Ge, and R–Ge are being built up, which were shown in La_2MGe_6, and in LuGe as well, to be still consistent with the Zintl-Klemm concept. For R^{III}Ge compounds, an additional feature arises, which makes them conceptually distinct, namely polycationic R_4 bonding, being an intermetallic extension to the classical GaSe case with 2c-2e Ga–Ga bonds.

Table S5. Bonding parameters for LuGe from position-space analysis (after PSC0 correction).

$Q^{eff}(Ge)$	ELIBON(Ge)	$N_{acc}^{ELI}(C^{Ge})$	$N_{val}^{ELI}(Ge)$	$N_{cbe}(Ge)$	$N_{lpe}(Ge)$
–1.34	–2.77	7.43	5.27	2.15	3.12

Atomic core charges corresponding to Ge^{4+} and Lu^{3+} have been selected. Q^{eff}(Ge) – effective QTAIM charge of the germanium atoms, the charge of lutetium has the opposite sign. ELIBON(Ge) – electron-localizability based oxidation number [39] of Ge. $N_{acc}^{ELI}(C^{Ge})$ – number of access electrons around the Ge core basin [40,41]. $N_{val}^{ELI}(Ge)$ – number of electrons in the ELI-defined valence shell of Ge [40,41]. $N_{cbe}(Ge)$ and $N_{lpe}(Ge)$ – numbers of electrons in covalent bonds and lone-pairs per Ge atom, respectively [40.41].

Table S6. Bonding parameters for LuGe from the position-space analysis.

ELI-D basin (B_i)	*Color in Figure 3, middle lower panel*	*Atomicity* Ge_kM_l	$\bar{N}(B_i)$	*cc*	*lpc*	$\sum_{j=1}^{k} p(B_i^{Ge_j})$	$\sum_{j=1}^{l} p(B_i^{Lu_j})$
Ge-Ge	yellow	Ge_2 (2*a*)	0.66	1.00	0.00	0.99	0.01
lp-Ge	red	$GeLu_4$ (5*a*)	2.25	0.48	0.52	0.76	0.24
lp-Ge	green	$GeLu_4$ (5*a*)	2.42	0.46	0.54	0.77	0.23
lp-Ge	orange	$GeLu_3$ (4*a*)	0.72	0.55	0.45	0.72	0.28
Lu_4	blue	Lu_4 (4*a*)	0.23	-	-	0.29	0.71

Data obtained after PSC0 correction referred to each ELI-D valence basin are listed. The atomicity of a basin denotes the number of atomic basins contributing to the population of the basin under consideration. $\bar{N}(B_i)$ the population of the ELI-D basin in electrons. *cc* and *lpc* – non-polar (covalent) character and polar (lone-pair) character of the ELI-D basin. $\sum_j^k p(B_i^{Ge_j})$ and $\sum_j^l p(B_i^{Lu_j})$ – the sums of the bond fractions of Ge and Lu, respectively, for the ELI-D basin [40.41].

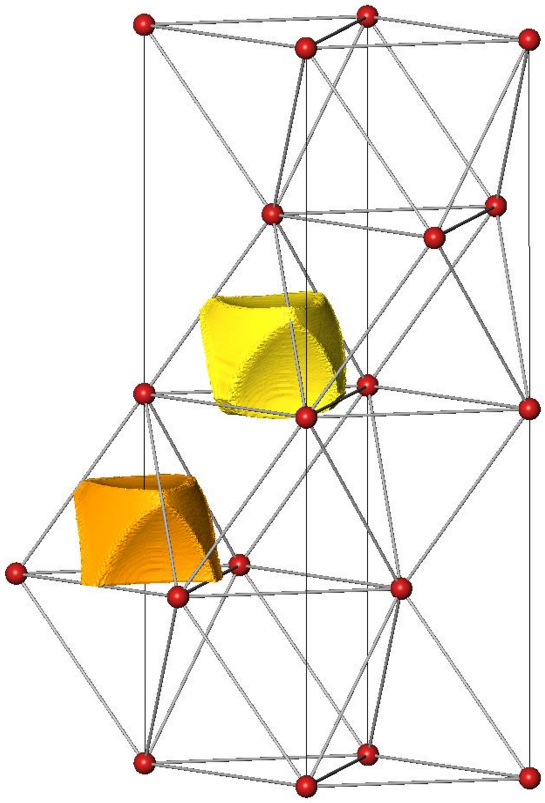

Figure S7. ELI-D bonding basins for the 4*a*-La_4 bonds in elemental lanthanum (Nd-type crystal structure).

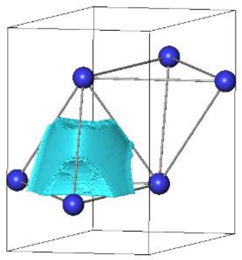

Figure S8. ELI-D bonding basin for 4*a*-Lu_4 bond in elemental lutetium (*hcp* Mg-type structure).

Bonding investigations on representatives of the α-TlI (CrB) and FeB structures types have the own history (e.g., [42-45]). Within the present study, qualitatively similar results as reported for LuGe(FeB) have been obtained also for LaGe(FeB), which is a good argument to expect it for all the remaining lanthanides with a magnetic moment corresponding to a formal 3+ state (NOT oxidation state). Moreover, hypothetical CaGe(FeB) (structure optimization) does not display 4-atomic Ca_4 ELI-D basins. The 4a-basin populations decrease along the series La > Lu > Ca. Consistent results are obtained when analyzing ELI-D in the hypothetical structures LaGe(α-TlI), LuGe(α-TlI), and CaGe(α-TlI). Here, only LaGe(FeB) displays a La_4 ELI-D basin, but with a reduced electron population. This indicates, that the α-TlI type is less favorable for the RE_4 type of bonding, such that even LuGe(α-TlI) fails to yield an Lu_4 ELI-D basin. Topological analysis reveals, that a tiny dominance of the density curvature in one direction causes this effect. Small changes of the optimized geometry or a different functional may already trunk this result and yield a Lu_4 ELI-D attractor as well. In order to verify this situation, the relative Laplacian of ELI-D has been used as an additional, softer and continuous measure for bond detection. A negative relative Laplacian of ELI-D inside the metal tetrahedra is found for LaGe(FeB)*, LuGe(FeB)*, LaGe(α-TlI)*, LuGe(α-TlI) which is necessarily the case only, where an ELI-D attractor is formed (indicated by asterisk '*'). In LuGe(α-TlI), CaGe(α-TlI) and CaGe(FeB), there are no bond critical points at the position of the possible R_4 bond attractor. They are shifted staying still inside the tetrahedron, and reveal a negative value for the relative Laplacian for LuGe(α-TlI) and positive values of the relative Laplacian for CaGe(α-TlI) and CaGe(FeB). The calculated relative Laplacian values at a point corresponding to the expected attractor position is clearly negative for LuGe(α-TlI) and scatter around zero for CaGe: slightly positive for CaGe(α-TlI) and slightly negative for CaGe(FeB). This marks a clear difference between "R^{III}Ge" and "M^{II}Ge" and supports the idea of a specific R_4 or M_4 bonding situation being more favorably supported by the FeB structure type (more degrees of freedom?) than in the α-ITl (CrB) type.

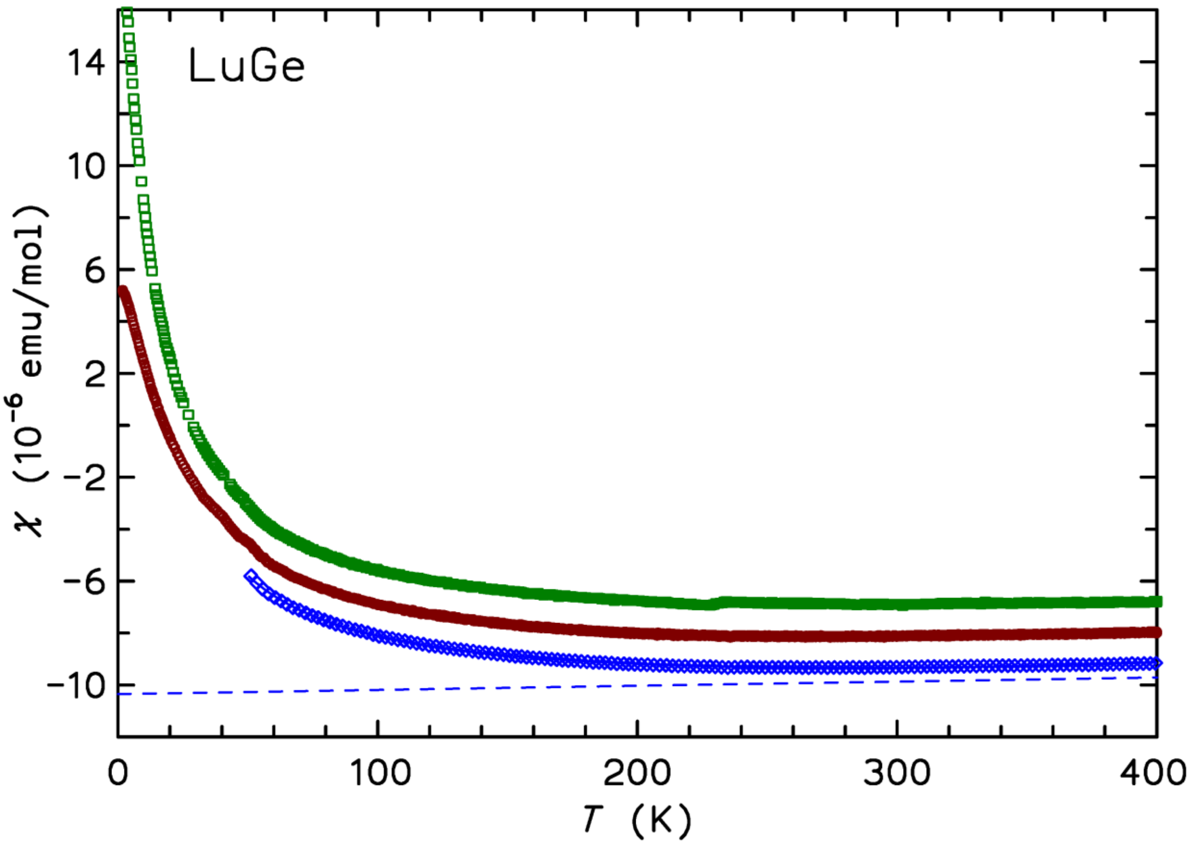

Figure S10. Temperature dependence of the magnetic susceptibility $\chi = M/H$ of a polycrystalline LuGe sample measured in two high magnetic fields, $\mu_0 H$ = 3.5 T (green) and 7.0 T (red). The difference of the susceptibility curves is due to traces of a ferromagnetic contamination (corresponding to the saturated magnetization of 1.5 ppm of metallic Fe). Correcting for this constant offset, the curve with the blue data points is obtained. These data can be fitted excellently with the relationship $\chi(T) = C/T + \chi_0 + \chi_1 T + \chi_2 T^2$. Here, C/T is a Curie-paramagnetic contribution (with C corresponding to 0.06 mol.% of $S = 1/2$ impurities), originating possibly from other magnetic rare-earth metal contaminations in the used Lu metal. The temperature-dependent term $\chi_1 T$ and $\chi_2 T^2$ are minor corrections and the finally derived temperature-independent susceptibility term χ_0 at $T = 0$ is -10(4) × 10^{-6} emu mol^{-1}. This diamagnetic value is of the order of the tabulated diamagnetic increment of the Lu^{3+} ion (-17 × 10^{-6} emu mol^{-1} [46]). Using this ionic value, the difference to the measured χ_0 of LuGe leaves some room for a small Pauli paramagnetic contribution (+7(4) × 10^{-6} emu mol^{-1}) from conduction electrons.

Additional measurements at low fields ($\mu_0 H$ = 0.5 mT and less; not shown) indicate the absence of bulk superconductivity with critical temperature T_c > 1.8 K in LuGe. However, a small diamagnetic signal indicates the presence of a superconducting minority phase (volume fraction of < 2 %) in the sample. The signal with onset at 3.3 K stems probably from the superconducting phase $LuGe_3$ [47].

References

[34] L. Akselrud, Yu. Grin. *J. Appl. Crystallogr.* **2014**, *47*, 803– 805.

[35] V. Blum, R. Gehrke, F. Hanke, P. Havu, V. Havu, X. Ren, K. Reuter, M. Scheffler, *Computer Phys. Comm*. **2009**, *180*, 2175-2196.

[36] J. P. Perdew, K. Burke, M. Ernzerhof, *Phys. Rev. Lett.*, **1996**, *77*, 3865-3868.

[37] M. Kohout, *Int. J. Quantum Chem*. **2004**, *97*, 651–658.

[38] M. Kohout, *Program DGrid-5.0*, Dresden, **2018**.

[39] I. Veremchuk, T. Mori, Yu. Prots, W. Schnelle, A. Leithe-Jasper, M. Kohout, Yu. Grin, *J. Solid State Chem*. **2008,** *181*, 1983-1991.

[40] D. Bende, F. R. Wagner, Yu. Grin, *Inorg. Chem.* **2015**, *54*, 3970-3978.

[41] F. R. Wagner, D. Bende, Yu. Grin. *Dalton Trans*. **2016**, *45*, 3236-3243.

[42] O. Bisi, L. Braicovich, C. Carbone, I. Lindau, A. Iandelli, G. L. Olcese, A. Palenzona, *Phys. Rev*. **1989**, *B40*, 10194–10209.

[43] A. Currao, J. Curda, R. Nesper, *Z. Anorg. Allg. Chem*. **1996**, *622*, 85–94.

[44] D. Becker, H. P. Beck. *Z. Kristallogr*. **2004**, *219*, 348–358.

[45] F. R. Wagner, Dissertation, Universitat Saarbrucken, **1993**.

[46] G. A. Bain & J. F. Berry, *J. Chem. Education* **2008,** *85*, 532-536.

[47] J.-M. Hübner, M. Bobnar, L. Akselrud, Y. Prots, Yu. Grin, U. Schwarz, *Inorg. Chem*. **2018**, *57*, 10295–10302.
